# Comparative RP-HPTLC Lipophilicity Analysis of Bioactive 1,3,4-Thiadiazole–Resorcinol Conjugates

**DOI:** 10.3390/ijms27198561

**Published:** 2026-09-25

**Authors:** Beata Paw, Marek Studziński, Sylwia Stec, Katarzyna Barańska, Irena Malinowska, Joanna Matysiak

**Affiliations:** 1 Department of Medicinal Chemistry, Medical University of Lublin, Jaczewskiego St. 4, 20-090 Lublin, Poland; sylwiastec66@gmail.com; 2 Department of Physical Chemistry, Faculty of Chemistry, Institute of Chemical Sciences, Maria Curie-Skłodowska University, 20-031 Lublin, Poland; marek.studzinski@umcs.pl (M.S.); irena.malinowska@umcs.pl (I.M.); 3 Independent Radiopharmacy Unit, Medical University of Lublin, Chodzki 4a, 20-093 Lublin, Poland; k.baranska955@gmail.com; 4 Department of Chemistry, University of Life Sciences in Lublin, Akademicka 15, 20-950 Lublin, Poland

**Keywords:** HPTLC, RP-18, RP-8, organic modifier, lipophilicity, correlation analysis, 1,3,4-thiadiazole-resorcinols

## Abstract

The behavior of bioactive 1,3,4-thiadiazoles of anticancer potency and cholinesterases (ChEs) inhibitors was examined by high-performance thin-layer chromatography (HPTLC) in a reversed phase (RP) system. The chromatographic systems consisted of C-18 or C-8-bonded silica as the stationary phase and binary mixtures of water with methanol, acetonitrile, acetone, or dioxane as the mobile phase. The lipophilicity parameters R_Mw_ (the R_M_ value of a solute extrapolated to pure water as the mobile phase) were determined based on the linear relationship between compound retention and water content in the mobile phase. R_Mw_ parameters obtained from various chromatographic systems were analyzed both in relation to each other and compared with other lipophilicity parameters, including log k_w_ values obtained by RP-18 HPLC and computationally calculated log P. Descriptive, correlation and principal component analyses were applied. The applicability of the HPTLC method for assessing the lipophilicity of 1,3,4-thiadiazole–resorcinol conjugates was demonstrated based on the log k_w_ values determined by HPLC according to the OECD-recommended procedure and described previously. The correlation coefficients (*r*) between the R_Mw_ parameters for these systems and log k_w_ (C-18) is ≥0.81, whereas for the C-8 stationary phases R_Mw_ versus log k_w_ is ≥0.95. Taking into account the results of descriptive and correlation analysis, the C18 stationary phase with methanol as the organic modifier and the C8 stationary phase with methanol or dioxan as the organic modifier were found to be the most suitable chromatographic systems for lipophilicity determination of bioactive compounds.

## 1. Introduction

Thin-layer (planar) chromatography (TLC) and high-performance liquid (column) chromatography (HPLC) are among the most commonly used chromatographic techniques [1,2]. Both methods are applied in normal-phase (NP) and reverse-phase (RP) variants. Chromatography with a hydrophobic stationary phase and a polar eluent (RP) currently plays an important role in both separating mixtures and determining selected physicochemical properties of the analyte, such as lipophilicity and dissociation constants. It is particularly useful for estimating the lipophilicity of biologically active compounds [2,3,4].

The International Union of Pure and Applied Chemistry (IUPAC) defines lipophilicity as the affinity of a molecule or moiety for a lipophilic environment [5]. Therefore, retention in a system with a hydrophobic octadecyl (C18) or octyl (C8) stationary phase and a polar mobile phase (water/organic modifier) provides a good model for determining lipophilicity. A system using pure water as the mobile phase would probably be even more effective. However, pure water cannot be used as the mobile phase for the vast majority of compounds, as it would significantly prolong the analytical process and produce very low or zero R_F_ values [6].

Lambert found that when the stationary phase is nonpolar, the retention of a compound in pure water is closely correlated with its lipophilicity [6]. Therefore, the Soczewiński–Wachtmeister equation was applied to determine the chromatographic lipophilicity parameters R_Mw_ (log k_w_) of compounds [7,8]. The equation describes a linear relationship between the retention parameter R_M_ and the volume fraction of the organic modifier ϕ in the binary mobile phase:

(1) R_M_ = R_Mw_ + Sϕ

where ϕ is the volume fraction of the organic modifier in the binary mobile phase; S is the slope of the regression equation; R_Mw_ is the intercept corresponding to 0% organic modifier content of the mobile phase [7]. The value of the lipophilicity parameter R_Mw_ are obtained by extrapolating to ϕ = 0 (i.e., 0% organic modifier content of the mobile phase). However, the extrapolated R_Mw_ values obtained using systems with different organic modifiers differ, sometimes substantially. The same trend is observed in HPLC. Despite the above, these values are well correlated and accurately reflect differences in lipophilicity among compounds tested under identical experimental conditions [9].

Methanol is recommended for binary water–organic component mobile phases as an organic modifier that is most similar to water, although acetonitrile (ACN) is also frequently used. Other organic solvents have also been tested for high-performance thin-layer chromatography (HPTLC), including propan-2-ol, propan-1-ol, tetrahydrofuran, dioxane, acetone, and ethanol [10,11].

Lipophilicity is a crucial physicochemical property of potential drug candidates and is considered at the early stages of drug design [12,13]. It determines pharmacokinetic processes, primarily by influencing passive transport across lipid membranes and the binding of compounds to blood proteins. Lipophilicity also affects pharmacodynamics by influencing ligand–target interactions. Therefore, drug-like molecules are expected to possess a specific lipophilic–hydrophilic balance, indicating a high probability of favorable ADME properties. RP chromatography (RP-8, RP-18), including HPTLC, remains a very important method for determining these properties [14].

HPTLC has been used to study the lipophilicity of commonly used drugs, cosmetic ingredients, food additives, and newly synthesized compounds with potential pharmaceutical applications. It has been applied to analyze structurally diverse cosmetic ingredients, such as sunscreens and preservatives, to predict drug-likeness and ADME properties [15]. Briciu et al. investigated artificial and natural sweeteners [16]. Other researchers have examined toxins, including mycotoxins and alkaloids [17]. The lipophilicity of the following drugs has also been analyzed by HPTLC: cephalosporins [18], xanthone derivative [13], and α-adrenergic and imidazoline receptor ligands [19]. Recent studies have explored the lipophilicity of selected non-steroidal anti-inflammatory drugs (NSAIDs) from the oxicam and coxib classes [20], selected neuroleptics [21], and several antimicrobial and immunosuppressive drugs [22]. In addition, TLC has been used to predict the hepatoselectivity of selected statins [23].

HPTLC is commonly applied to investigate the lipophilicity of newly synthesized compounds, including imidazoline derivatives [24], 1-acridine and quinoline derivatives as acetylcholinesterase inhibitors [25], betulin derivatives [26], heterocyclic-1,3,4-thiadiazole hybrids [27], dipyridothiazine dimers with anticancer activity [28], thiazolyl-1,3,4-oxadiazole derivatives with antifungal activity [29], N1-arylidene-thiosemicarbazones and 1,3,4-thiadiazolines with antimicrobial activity [10], and other compounds [29].

Comparative studies conducted by our research team on the lipophilicity of 5-heterocyclic 2-(2,4-dihydroxyphenyl)-1,3,4-thiadiazoles, determined by HPLC and HPTLC, showed a high correlation between the log k_w_ (HPLC) and R_Mw_ (HPTLC) parameters. Particularly favorable characteristics as organic modifiers in the HPTLC assessment of lipophilicity for this group of compounds were observed for the commonly used solvents methanol and dioxane [27]. We decided to extend our chromatographic investigations to another group of compounds, namely 5-alkyl and aryl 2-(2,4-dihydroxyphenyl)-1,3,4-thiadiazoles [30,31]. HPLC data for this group of compounds using different stationary phases that mimic biological systems, including C8, C18, Immobilized Artificial Membrane (IAM), and Chol (with bonded cholesterol molecules), have been previously reported [32]. Furthermore, the distribution coefficient (log D_7.4_) values for the compounds were determined from retention data obtained using the C18/methanol system in accordance with the OECD-recommended procedures (The Organization for Economic Co-operation and Development).

The present paper reports the results obtained for this group of compounds using planar chromatography HPTLC. The aim of this study was to compare the retention parameters of compounds obtained using HPTLC systems with two hydrophobic stationary phases and four different organic modifiers in order to identify the optimal HPTLC system for lipophilicity determination and separation. The obtained lipophilicity parameters (R_Mw_) were compared with those calculated using computational methods, log P (the log of the octanol/water partition coefficient P of a solute). To provide a more comprehensive evaluation, the results were also compared with the lipophilicity parameters (log k_w_) obtained using an HPLC system, which is recommended by the OECD for the chromatographic determination of lipophilicity [33]. In the present study, HPLC-derived data (log k_W_ values), obtained mainly from the C18 and additionally from the C8 systems, were used to demonstrate the equivalence of lipophilicity parameters determined by HPTLC and HPLC methods, and to confirm the applicability of HPTLC technique in various chromatographic systems for the assessment of the lipophilicity of bioactive compounds.

## 2. Results

### 2.1. Compounds Studied

The evaluated compounds were 5-alkyl- and aryl-substituted 2-(2,4-dihydroxyphenyl)-1,3,4-thiadiazoles. In some derivatives, these substituents are linked to the heterocyclic ring by an -NH- or -CH_2_O-group, which increases molecular flexibility. Certain derivatives also contain an Et or Cl substituent in the 2,4-dihydroxyphenyl moiety, which increases their lipophilicity [30,31]. The structure of the tested compounds is presented in Figure 1.

### 2.2. Determination of R_Mw_ Parameters

Chromatographic analyses were conducted using HPTLC. Two hydrophobic phases with varying degrees of hydrophobicity were used: octyl (C8), which is less hydrophobic, and octadecyl (C18), which is more hydrophobic. Four organic modifiers were used in the binary water/organic mobile phase: methanol (Met), acetone (ACE), acetonitrile (ACN), and dioxane (Dx). For all compounds in the eight studied systems, linear relationships were found between the R_M_ values and the content of the organic modifier (ϕ) in the mobile phase (Equation (1)). The values of the correlation coefficient *r* were lowest in the systems containing ACE as the organic modifier. The R_Mw_ values for all systems were obtained using Equation (1) and the extrapolation method (Tables S1–S8, Supplementary Materials). The retention parameters (R_Mw_) of the investigated compounds in different chromatographic systems are compared in Figure 2.

In general, for the C18 stationary phase, the lowest retention among the tested mobile phases was observed when ACN was used as the organic modifier, whereas the highest retention was obtained with Met. In most cases, the systems containing Dx and ACE yielded intermediate R_Mw_ values between those obtained with Met and ACN. The R_Mw_ values obtained with ACE were generally higher than those found with Dx. Compound **11** containing an -OCH_2_-linker and an -OCF_3_ substituent was an exception, for which retention was highest when Dx was used as the mobile phase component.

This might be explained by the greater conformational flexibility of the molecule, which can significantly affect intermolecular interactions and alter chromatographic selectivity. A similar general trend for R_Mw_ values was observed when the C8 stationary phase was used. However, the described anomaly did not occur in this case. Instead, a decrease in retention was noted for compounds **8** and **12**. This may be the result either of the formation of mesmeric (e.g., -OCH_3_ substituent) or tautomeric (-NH-substituted thiadiazole ring) structures, characterized by a different charge distribution and degree of hydratation, thereby affecting its affinity for the stationary phase. These processes will also depend largely on the environment (aqueous-organic mixture) in which the compound is located.

### 2.3. Descriptive Statistics of R_Mw_ Parameters

A comparison of the distribution of R_Mw_ values for the investigated compounds (descriptive statistics) revealed the highest mean R_Mw_ value for the RP-18/Met system and the lowest mean R_Mw_ value for the RP-18/ACN system (Table 1), which is connected with the electron-acceptor contribution to the interaction components [34]. A comparison of the mean retention values for the same mobile phases on the RP-8 stationary phase revealed smaller differences, which may be attributed to the effect of stationary phase hydrophobicity on retention.

### 2.4. Correlation Analysis

The correlation coefficients (*r*) between parameters R_Mw_ and S for individual chromatographic systems are presented in Table 2. The values of *r* ranged from −0.8623 to −0.9958 and exceeded 0.99 for most systems. The lowest correlation coefficients were obtained for the systems using ACN as the mobile phase. The negative values of the correlation coefficients (*r*) are expected, as they reflect the inherent inverse linear relationship between the lipophilicity intercept (R_Mw_) and the slope (S), which is always negative in reversed-phase systems.

The values of lipophilicity parameters R_Mw_ obtained for individual chromatographic systems were compared by correlation analysis (Table 3). For the most commonly used mobile phases containing Met and ACN, the relationships between R_Mw_ parameters for individual stationary phases are expressed by the following equations:

(2) R_Mw_ C18/Met = 1.0069 (±0.3364) + 1.2150 (±0.1333) R_Mw_ C18/ACN

n = 18, R = 0.9157, R^2^ = 0.8365, $R_{adj}^{2}$ = 0.8264, F(1,16) = 83.07, *p* = 0.0000, s = 0.2538

(3) R_Mw_ C8/Met = 0.1630 (±0.3801) + 1.3468 (±0.1399) R_Mw_ C8/ACN

n = 18, R = 0.9234, R^2^ = 0.8527, $R_{adj}^{2}$ = 0.8435, F(1,16) = 83.07, *p* = 0.0000, s = 0.2539

Correlation coefficients (*r*) ranged from 0.9118 to 0.9826 in a comparison of the R_Mw_ values obtained with the C8 and C18 stationary phases using the same organic modifiers (Table 3). The values of *r* were highest for ACN and Dx as organic modifiers (Equations (4) and (5)):

(4) R_Mw_ C18/ACN = −0.2933 (±0.1329) + 1.0349 (±0.0489) R_Mw_ C8/ACN

n = 18, R = 0.9826, R^2^ = 0.9655, $R_{adj}^{2}$ = 0.9633, F(1,16) = 447.16, *p* = 0.0000, s = 0.0885

(5) R_Mw_ C18/Dx = 0.2914 (±0.2394) + 1.0161 (±0.0735) R_Mw_ C8/Dx

n = 18, R = 0.9606, R^2^ = 0.9227, $R_{adj}^{2}$ = 0.9179, F(1,16) = 190.99, *p* = 0.0000, s = 0.2126

The slopes of Equations (4) and (5) are similar and approximately equal to 1. This indicates that the R_Mw_ values obtained using ACN and Dx as organic modifiers differ between individual compounds by a specific amount. The low intercept indicates that these differences are small. In case of Dx (Equation (5)), these values are higher for the C18 phase than for the C8 phase. No similarly fitting equations were obtained for other systems. For the C18 phase, the strongest correlation between lipophilicity parameters R_Mw_ was observed in systems where Met and ACN were used as organic modifiers (Equation (2)). For the C8 phase, the strongest correlation between these parameters was found in the systems using Met and Dx; however, these differences were not statistically significant (Table 3, Equation (6)).

(6) R_Mw_ C8/Met = 1.0762 (±0.2725) + 0.8481 (±0.0837) R_Mw_ 8/Dx

n = 18, R = 0.9301, R^2^ = 0.8652, $R_{adj}^{2}$ = 0.8567, F(1,16) = 102.67, *p* = 0.0011, s = 0.2420

A comparison of the correlations between individual stationary phases demonstrated the strongest correlation between the lipophilicity parameters obtained with ACE as the organic modifier (Table 3). For the C8 phase, the highest correlation was noted for the ACN system (Equation (7)). For the C18 phase, the corresponding result was obtained for the Dx system, although the correlation was slightly weaker (Equation (8)).

(7) R_Mw_ C8/ACE = −0.9671 (±0.2414) + 1.6534 (±0.0889) R_Mw_ C8/ACN

n = 18, R = 0.9777, R^2^ = 0.9558, $R_{adj}^{2}$ = 0.9539, F(1,16) = 346.07, *p* = 0.0000, s = 0.1607

(8) R_Mw_ C18/ACE = 0.0383 (±0.3135) + 0.9830 (±0.0871) R_Mw_ C18/Dx

n = 18, R = 0.9425, R^2^ = 0.8884, $R_{adj}^{2}\;$= 0.8814, F(1,16) = 127.35, *p* = 0.0000, s = 0.2664

The slope and the intercept of Equation (8) indicate that the R_Mw_ values obtained for both systems are highly similar.

A comparison of all the chromatographic systems revealed that the R_Mw_ values obtained for the C8/ACE system were most strongly correlated with those obtained for the C8/ACN (Equation (8)) and C8/Dx (Equation (9)) systems.

(9) R_Mw_ C8/ACE = 0.2108 (±0.2163) + 1.0234 (±0.0665) R_Mw_ C8/Dx

n = 18, R = 0.9679, R^2^ = 0.9368, $R_{adj}^{2}$ = 0.9329, F(1,16) = 237.18, *p* = 0.0000, s = 0.1913

The slope and intercept of Equation (9) indicate that the R_Mw_ values obtained using ACE and Dx are quite similar. Similar results were observed for the C18 stationary phase with the same organic modifiers (Equation (8)). These findings are consistent with the results of descriptive statistics.

High correlations were also observed between the R_Mw_ values obtained for the C18/ACN system and those obtained using the C8 stationary phase. The correlation coefficients ranged from 0.9358 for Met to 0.9826 for ACN (Table 3, Equations (4), (10) and (11)).

(10) R_Mw_ C8/ACE = −0.3321 (±0.3060) + 1.5307 (±0.1213) R_Mw_ C18/ACN

n = 18, R = 0.9533, R^2^ = 0.9087, $R_{adj}^{2}$ = 0.9030, F(1,16) = 159.31, *p* = 0.0000, s = 0.2309

(11) R_Mw_ C8/Dx = −0.3801 (±0.3132) + 1.4351 (±0.1241) R_Mw_ C18/ACN

n = 18, R = 0.9450, R^2^ = 0.8931, $R_{adj}^{2}$ = 0.8864, F(1,16) = 133.66, *p* = 0.0000, s = 0.2363

To provide a more comprehensive evaluation, the obtained data were also compared with those previously reported for the tested group of compounds using HPLC, including the C18/Met system recommended by the OECD for the chromatographic determination of lipophilicity (Table S9, Supplementary Materials) [32,33]. The R_Mw_ value for the C18/Met system showed the strongest correlation with the lipophilicity parameter log k_w_ determined by HPLC using the C18/Met system. This relationship is described by the following equation (Figure 3a):

(12) R_Mw_ C18/Met = 1.0970 (±0.3454) + 0.9757 (±0.1132) log k_w_ C18

n = 18, R = 0.9070, R^2^ = 0.8227, $R_{adj}^{2}$ = 0.8116, F(1,16) = 74.22, *p* = 0.0059, s = 0.2859

Interestingly, the slope of Equation (12) is close to 1, indicating that the log k_w_ and R_Mw_ parameters for individual compounds differ by a constant value resulting solely from the use of HPLC instead of TLC. The log k_w_ parameter obtained for the C8/Met system showed the strongest and comparable correlations with the R_Mw_ values for the C8/Dx and C8/Met systems. In the latter case, the following equation was obtained (Figure 3b):

(13) R_Mw_ C8/Met = −0.0819 (±0.3165) + 0.8757 (±0.0710) log k_w_ C8

n = 18, R = 0.9512, R^2^ = 0.9048, $R_{adj}^{2}$ = 0.8989, F(1,16) = 152.09, *p* = 0.0059, s = 0.203

The lipophilicity descriptors obtained by HPTLC were also compared with the log k_w_ parameter estimated using the IAM/Met HPLC system. The values of Pearson correlation coefficients were high, especially for the systems using Met as the organic modifier, for both stationary phases (*r* = 0.88). These correlation coefficients are similar to those obtained for the C18/Met HPLC system [32].

### 2.5. Principal Component Analysis

Principal Component Analysis (PCA) was performed to make deeper insight into relations among experimentally calculated R_Mw_ of the tested compounds in RP system by TLC. Analysis was performed using orthogonal coordinates and quartilmax rotation method in order to better analyze dependencies among obtained retention lipohilicity descriptors (Tables S10 and S11, Figure S1, Supplementary Materials). The score plot after this operations was presented in Figure 4. In the center of the graph, compounds **2**, **3**, **4** and **13** can be seen, what suggests that their chromatographic behavior is most similar to each other, regardless of stationary and mobile phase used parameters. The presence of F or Cl atom in *meta* position of the benzene ring (compounds **3** and **4**) does not induce as strong retention changes as those located in *para* position (**5** and **6**). On the far left side, compounds with HO- groups are present (**7**, **8**), their separation from the other points may be interpreted that, in their case, effect of residual silanol groups interactions influences their chromatographic behavior. To the right, points of unsubstituted compounds (**1**, **12**) can be observed. This position implicates smaller effect of residual silanol groups on their retention comparing to compounds **7** and **8**. In the upper right quarter of the graph, compounds **5**, **6**, **10**, **11** and **17** are located. All of them possess strong electron-withdrawing substituents (Cl-, F- or F_3_C-) in the *para* position of the benzene ring. These enhance the hydrophobicity of the molecules and significantly influences their retention in the chromatographic systems studied, in contrast to the compounds located near the center of the plot. Over the lower right and the upper left quarters of the graph, 2-aminoderivatives are located, forming the straight line (except **17** and **16** with-F and -Cl atom, respectively what depicts similar character of this compounds (**12**, **13**, **14**, **15** and **18**). and regular changes in the lipophilicity among this homolog compounds.

## 3. Discussion

Very good linear relationships between the volume fraction of organic modifier and the R_M_ values were obtained for both stationary phases. Slightly poorer regression statistics were observed for the system with ACE as the organic modifier.

In general, for both stationary phases, retention was lowest when ACN was used as the organic modifier, and it was highest when Met was used. In most cases, the systems containing Dx and ACE yielded intermediate R_Mw_ values between those obtained with Met and ACN. Similar relationships were observed for 5-heterocyclic 2-(2,4-dihydroxyphenyl)-1,3,4-thiadiazoles [27]. In studies where the lipophilicity of NSAIDs was examined with the use of Met and ACE, significantly higher R_Mw_ values were obtained with Met as the organic modifier on both C18 and C8 stationary phases [20]. In another study involving the same group of drugs, the highest R_Mw_ values were also reported with Met, whereas the values observed with ACN were not always lower than those obtained with Dx and ACE [37].

The widest range of R_Mw_ values for the studied compounds was achieved when Met or ACE were applied as the organic modifier on both stationary phases. In this system, different substituents of the tested analytes had the greatest impact on retention, reflecting its high selectivity and resulting in the greatest diversity of the obtained R_Mw_ parameters. This is confirmed by the range and standard deviation values. The R_Mw_ values obtained using ACN as the organic modifier covered a significantly narrower range. The range of R_Mw_ values was wider for the C18 phase than for the C8 phase or was comparable in the case of Dx. Similar results were reported when NSAIDs were tested [20]. In a study examining dihydroxythiobenzanilides, the selectivity of the C18 system increased in the following order: ACN < ACE < Met, which is consistent with the present findings [38].

The influence of the dipole moment of organic modifiers on the value, range, or dispersion of R_Mw_ could not be explained (µ: Dx, 0.45 D; water, 1.87 D; ACE, 2.69 D; Met, 2.87 D; ACN, 3.44 D) [39]. The systems using ACN, which has the highest dipole moment, are characterized by the lowest R_Mw_ values covering the narrowest range. Therefore, changes in the lipophilicity of structurally similar analogues, determined using ACN as the organic mobile phase modifier, may be small, which can complicate interpretation because lipophilicity parameters may not be sufficiently differentiated.

The obtained R_Mw_ values generally differed across the organic solvents, although they were relatively highly correlated. The R_Mw_ values were most similar for the mobile phases containing ACE and Dx on both stationary phases.

The HPTLC results were compared with those obtained by HPLC using the C18/Met system, which is recommended by the OECD for the chromatographic assessment of compound lipophilicity [33]. Among the systems based on the C18 stationary phase, log k_w_ showed the strongest correlation with the R_Mw_ values obtained using Met. On the other hand, the R_Mw_ values obtained from the C18/Met and C18/ACN systems were most closely correlated within the same stationary phase. In another study using a C18 stationary phase, Met was also identified as the preferred modifier for lipophilicity estimation; Dx gave similar results, but ACN performed significantly worse [11]. These findings could be attributed to the fact that the cited study analyzed a set of structurally diverse compounds.

For the C8 stationary phase, the values of R_Mw_ obtained with all organic modifiers showed comparable correlations with the log k_w_ values determined in the C18/Met HPLC system. The correlation coefficient (*r*) was slightly higher when Dx was used as the organic modifier. However, the correlation was comparable to that obtained with Met, which is the recommended and most commonly used organic modifier. Another study also indicated dioxane as a favorable organic modifier [27]. The R_Mw_ values obtained on the C8 stationary phase were highly correlated with the log k_w_ values determined in the C8/Met HPLC system. As expected, the strongest correlation was observed when Met was used as the organic modifier.

In addition, the R_Mw_ values obtained from all C8 systems were correlated with both log k_w_ IAM and the calculated partition coefficient, log P Cons. The highest correlation coefficient (*r*) was obtained for R_Mw_ from the system containing Dx.

The high correlation coefficients for the above relationships, the relatively high water content that can be used in the Dx–water mobile phase for retention studies, the high *r* values for the R_Mw_ versus φ regressions, and the wide range of R_Mw_ values obtained indicate that Dx may serve as an alternative solvent for assessing the lipophilicity of the investigated compounds in a system with a C8 stationary phase.

These findings indicate that the log k_w_ and R_Mw_ parameters may be similarly useful for assessing the lipophilicity of the investigated bioactive compounds.

Beside of the obvious differences among HPLC and HPTLC methods, applying the proposed HPTLC allows for investigation of a larger number of substances in the shorter time, utilizing more accessible instruments comparing to HPLC. without significant worsening of quality of data obtained. HPTLC gives the ability of acquiring full UV spectrum of investigated compounds regardless of the organic solvent used as mobile phase modifier or sample solvent. Moreover, the method is also more environmentally friendly comparing to HPLC due to the lower organic solvent and energy consumption. The main disadvantages of HPTLC are unequilibrated conditions of chromatogram development (stationary phase wetting and chromatogram development takes place in the same time) what results in worse separation efficiency and repeatability of obtained results in comparison to HPLC.

## 4. Materials and Methods

### 4.1. Compounds

The studied compounds (**1**–**18**) were available in our laboratory at the Department of Chemistry, University of Life Sciences in Lublin. They were previously identified as acetylcholinesterase (AChE) and butyrylcholinesterase (BuChE) inhibitors [30] and as agents with antiproliferative activity against human cancer cell lines [31]. They were synthesized by treating commercially available hydrazides or thiosemicarbazides with sulfinylbis[(2,4-dihydroxyphenyl)methanethione] (STB) or its ethyl or chlorine analogues [40].

### 4.2. HPTLC

Chromatographic analyses were performed by horizontal chamber TLC using HPTLC 10 × 10 cm plates coated with octadecyl- or octyl-modified silica (Merck, Darmstadt, Germany). The tested substances were dissolved in Met to obtain solutions with a concentration of 1 mg/mL. Aliquots (2 µL) of the test solutions were applied to the chromatographic plates at the starting line located 1 cm from the plate edge. An TLC developing horizontal chamber (DS L, Chromdes, Lublin, Poland) was used. The plates were developed over a distance of 9 cm. The mobile phases consisted of water and the following organic modifiers: Met, ACN, ACE, or Dx. The organic solvent in the binary mobile phases had the following composition: RP-18 F_254_ phase: Met: 50–90%; ACN: 40–90%; ACE: 50–80%; and 1,4–Dx: 40–70%; RP-8 F_254_ phase: Met: 50–90%; ACN: 40–80%; ACE and 1,4-Dx: 40–70%. Spectral-grade solvents were purchased from Merck (Darmstadt, Germany). After chromatographic development, the plates were dried at room temperature. Next, the spots were visualized at 254 nm (excitation of the fluorescent indicator) and 366 nm (absorption by aromatic structures) using an analytical UV lamp (Haaland HA-05, Warsaw, Poland). All experiments were performed at ambient temperature (22 ± 2 °C). The R_F_ values (retention factors) were calculated as follows: R_F_ = a/b, where *a* s the migration distance of the compound (mm), and *b* is the migration distance of the mobile phase (mm). The obtained R_F_ values were converted to R_M_ values according to the Bate-Smith and Westall equation: R_M_ = log[(1/R_F_) − 1], where R_M_ is the TLC equivalent of the log *k* parameter used in column chromatography [41].

### 4.3. Computational Methods

Log P values were calculated using ChemDraw v. 10.0 [35]. Regression, descriptive and correlation analyses were conducted using Statistica v. 7.1 [42] and JASP v. 17.2.1 [43].

## 5. Conclusions

The R_Mw_ chromatographic parameters determined by HPTLC under the tested conditions can be successfully used to describe the lipophilicity of the analyzed 1,3,4-thiadiazoles.

Studies have revealed that Met is the preferred organic modifier for evaluating the lipophilicity of compounds using the C18 stationary phase. Although ACN offers some favorable characteristics, Met is generally considered a better option because, among other factors, it provides a wider range of R_Mw_ values.

For the C8 stationary phase, Met is also a suitable organic modifier, although equally satisfactory results were obtained using dioxane.

The R_Mw_ values obtained using ACN or dioxane as organic modifiers were the most similar to those determined on both stationary phases.

HPTLC may be applied as an alternative to HPLC for assessing the lipophilicity of thiadiazole–resorcinol conjugates. This reliable and simple technique may be particularly useful when access to the relatively expensive HPLC instrumentation is limited. However, commonly recognized limitations, such as signal broadening and poorer repeatability compared to HPLC, must be taken into account.

The observed behavior in the chromatographic systems studied may be applicable only to compounds structurally similar to those investigated. Therefore, it would be of interest to perform similar chromatographic analyses on compounds with entirely different structures and properties. Future research will also include testing other chromatographic systems, such as medium-polarity stationary phases in the RP system. Another research direction will involve correlating HPTLC chromatographic descriptors with the biological activity of compounds and exploring their potential use in predicting ADME parameters.

## Figures and Tables

**Figure 1:**
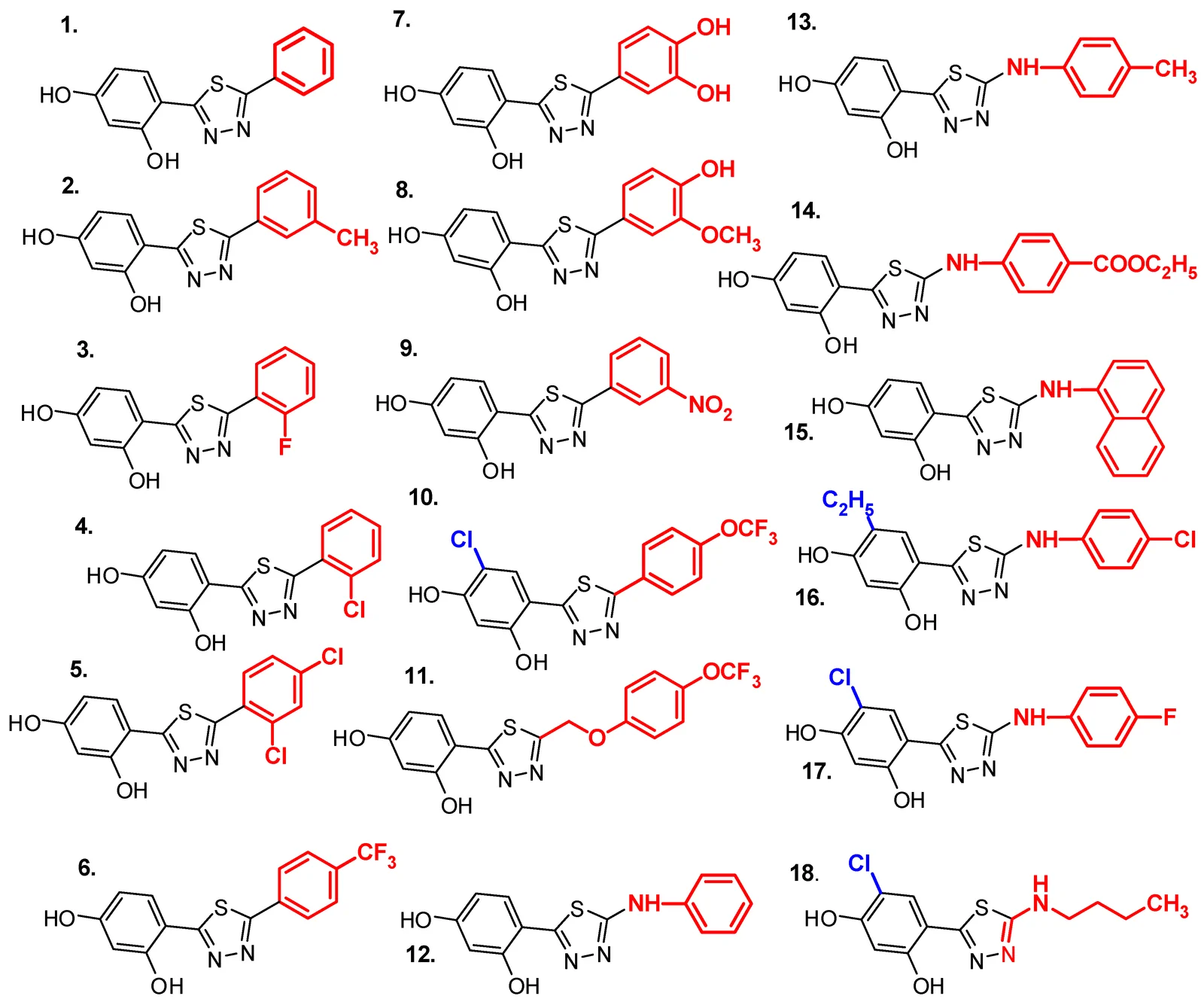
Structure of 5-modified 2-(2,4-dihydroxyphenyl)-1,3,4-thiadiazoles (**1**–**18**).

**Figure 2:**
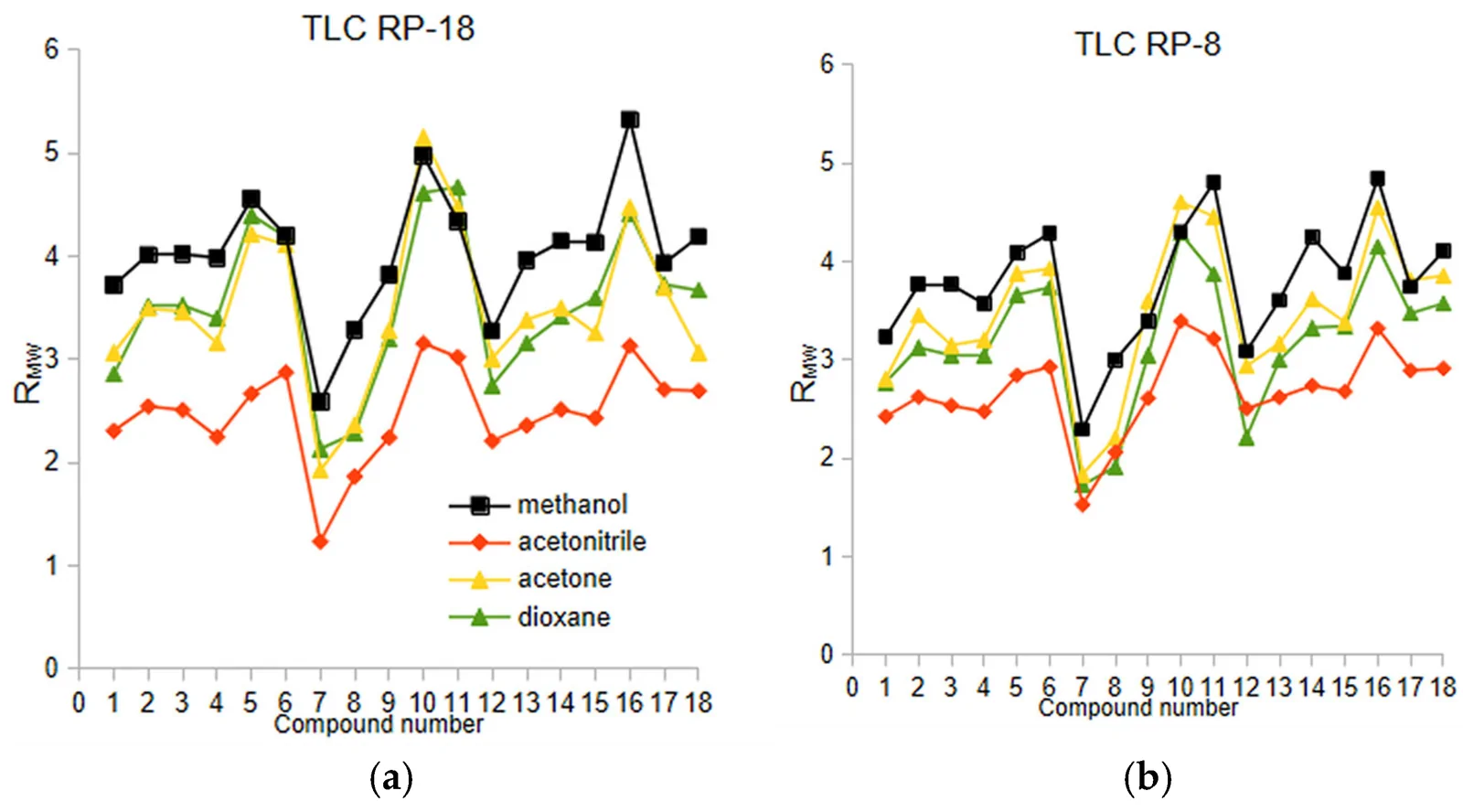
R_MW_ values calculated from experiments performed on RP-18 HPTLC (**a**) and RP-8 HPTLC (**b**).

**Figure 3:**
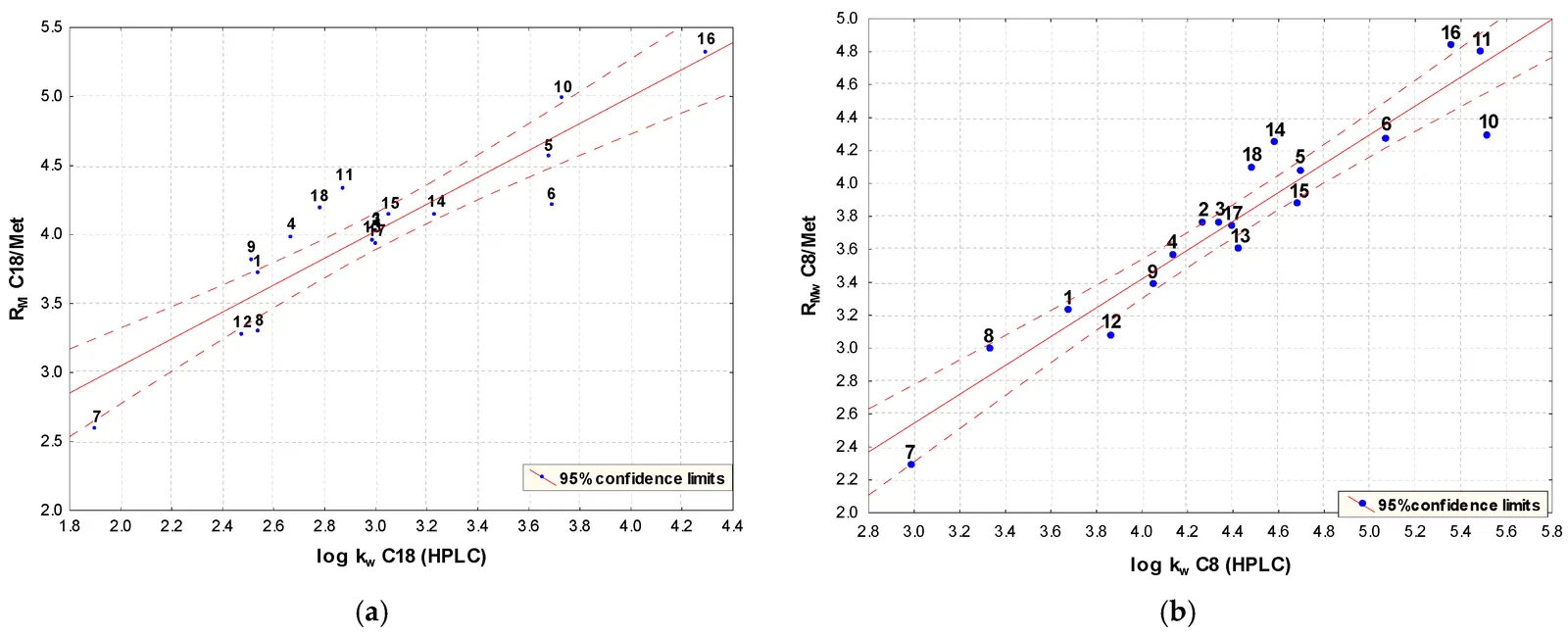
Relationships between the values of parameters log k_w_ (HPLC) and R_Mw_ (HPTLC) obtained using C18/Met (**a**) and C8/Met (**b**) systems, respectively.

**Figure 4:**
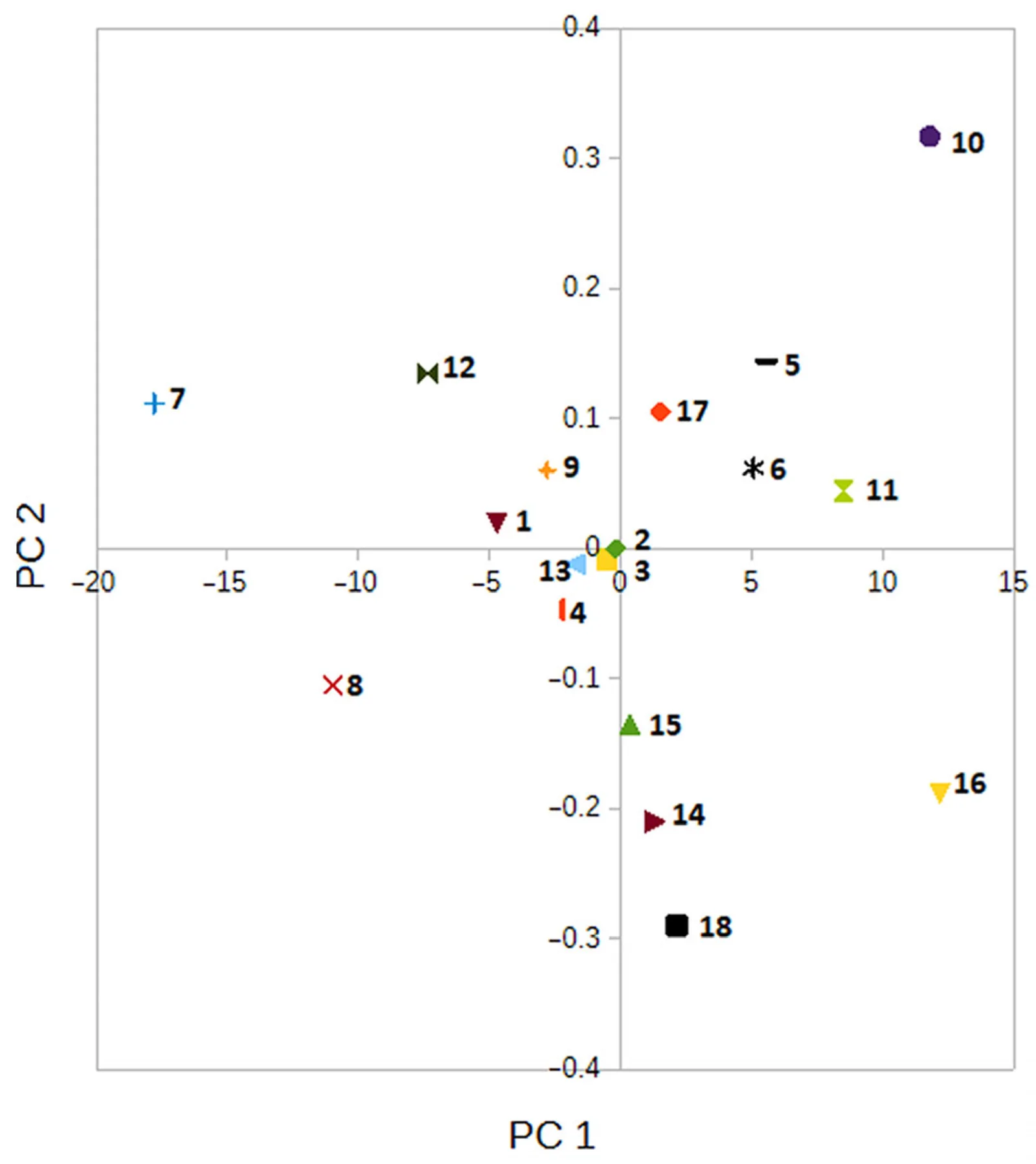
Score plot of components obtained for experimental data PCA.

**Table 1:** Descriptive statistics of R_Mw_ parameters of the investigated compounds in various chromatographic systems.

Param.	R_Mw_ C18/Met	R_Mw_ C18/ACN	R_Mw_ C18/ACE	R_Mw_ C18/Dx	R_Mw_ C8/Met	R_Mw_ C8/ACN	R_Mw_ C8/ACE	R_Mw_ C8/Dx
Mean ^1^	4.024	2.483	3.505	3.526	3.776	2.683	3.469	3.184
Std. Dev. ^2^	0.613	0.462	0.774	0.742	0.639	0.438	0.741	0.701
Minimum ^3^	2.585	1.234	1.926	2.133	2.292	1.529	1.837	1.727
Maximum ^3^	5.322	3.154	5.156	4.669	4.84	3.392	4.602	4.283
Range ^4^	2.737	1.92	3.23	2.536	2.548	1.863	2.765	2.556
Spread ^5^	2.059	2.556	2.677	2.189	2.112	2.218	2.505	2.48

^1^ Arithmetic mean of R_Mw_ values calculated for all compounds in the given chromatographic system. ^2^ Standard deviation of R_Mw_ values in the given system. ^3^ The lowest and highest R_Mw_ values, respectively, in the given system. ^4^ The difference between the highest and the lowest R_Mw_ values in the given system. ^5^ The ratio of the maximum to the minimum R_Mw_ value in the given chromatographic system.

**Table 2:** Correlation coefficients (*r*) between parameters R_Mw_ and S for individual chromatographic systems.

System	C18/Met	C18/ACN	C18/ACE	C18/Dx	C8/Met	C8/ACN	C8/ACE	C8/Dx
*r*	−0.9843	−0.8623	−0.9935	−0.9958	−0.9907	−0.9213	−0.9938	−0.9929

**Table 3:** Correlation coefficients (*r*) between the R_Mw_ values obtained by HPTLC, calculated log P values, and log k_w_ obtained by HPLC.

System	R_Mw_ C18/Met	R_Mw_ C18/ACN	R_Mw_ C18/ACE	R_Mw_ C18/Dx	R_Mw_ C8/Met	R_Mw_ C8/ACN	R_Mw_ C8/ACE	R_Mw_ C8/Dx
R_Mw_ C18/Met	1.0000							
R_Mw_ C18/ACN	0.9157	1.0000						
R_Mw_ C18/ACE	0.8916	0.9261	1.0000					
R_Mw_ C18/Dx	0.8970	0.9275	0.9425	1.0000				
C8/Met	0.9118	0.9358	0.8591	0.9220	1.0000			
R_Mw_ C8/ACN	0.9154	0.9826	0.9255	0.9180	0.9234	1.0000		
R_Mw_ C8/ACE	0.9080	0.9533	0.9264	0.9493	0.9273	0.9777	1.0000	
R_Mw_ C8/Dx	0.9414	0.9450	0.9160	0.9606	0.9301	0.9470	0.9679	1.0000
Log k_w_ C18 ^1^	0.9070	0.8223	0.8417	0.8097	0.8189	0.8073	0.8116	0.8715
Log k_w_ IAM ^1^	0.8825	0.7787	0.8186	0.8671	0.8852	0.7988	0.8609	0.9485
Log k_w_ C8 ^1^	0.9021	0.9394	0.9355	0.9555	0.9512	0.9461	0.9479	0.9523
Clog P ^2^	0.8080 (**12**) ^4^	0.7765 (**15**) ^4^	0.7391 (**15**) ^4^	0.7061 (**15**) ^4^	0.7950 (**11**) ^4^	0.8052 (**15**) ^4^	0.7595	0.9157 (**18**) ^4^
Log P ^2^	0.8465	0.8494	0.8988	0.8772	0.8184	0.8926	0.9330 (**18**) ^4^	0.8772
Log P Cons ^3^	0.8841	0.8365	0.9139	0.8799	0.8059	0.8546	0.8490	0.9403

^1^ Log k_w_ values taken from Ref. [32]. ^2^ Log P—calculated using ChemDraw [35]. ^3^ Log P Cons calculated using Marvin v. 19.9 software [36] and taken from Ref. [32]. ^4^ Outlier compound.

## Data Availability

The original contributions presented in this study are included in the article/Supplementary Materials. Further inquiries can be directed to the corresponding author.

## Supplementary Materials

The following supporting information can be downloaded at https://www.mdpi.com/article/10.3390/ijms27198561/s1.
