# Supplementary material for: Comparative RP-HPTLC Lipophilicity Analysis of Bioactive 1,3,4-Thiadiazole–Resorcinol Conjugates
Source: Int J Mol Sci. 2026 Sep 25;27(19):8561. doi: 10.3390/ijms27198561 (PMC13665130; doi:10.3390/ijms27198561)
Supplement: Supplementary file 1 [file ijms-27-08561-s001.zip › ijms-4529972-supplementary.pdf]

Comparative RP-HPTLC Lipophilicity Analysis of Bioactive 1,3,4-Thiadiazole–Resorcinol Conjugates

Beata Paw [1,*], Marek Studziński [2], Sylwia Stec [1], Katarzyna Barańska [3], Irena Malinowska [2], and Joanna Matysiak [4,*]

1 Department of Medicinal Chemistry, Medical University of Lublin, Jaczewskiego St. 4, 20-090 Lublin, Poland; sylwiastec66@gmail.com

2 Department of Physical Chemistry, Faculty of Chemistry, Institute of Chemical Sciences, Maria Curie-Skłodowska University, 20-031 Lublin, Poland; marek.studzinski@umcs.pl (M.S.); irena.malinowska@umcs.pl (I.M.)

3 Independent Radiopharmacy Unit, Medical University of Lublin, Chodzki 4a, 20-093 Lublin, Poland; k.baranska955@gmail.com

4 Department of Chemistry, University of Life Sciences in Lublin, Akademicka 15, 20-950 Lublin, Poland

* Correspondence: beata.paw@umlub.edu.pl (B.P.); joanna.matysiak@up.edu.pl (J.M.); Tel.: +48-814487389 (B.P.); +48-814456816 (J.M.)

Table S1. R_{Mw}, S parameters and statistics[1] obtained by C-18/methanol HPTLC.

No.	R_{Mw}	$S(R_{Mw})$	S	S(S)	r	Se
**1.**	3.7149	0.1617	-4.6025	0.2264	-0.9964	0.0716
**2.**	4.0102	0.1038	-4.7565	0.1454	-0.9986	0.046
**3.**	4.0213	0.0947	-4.7727	0.1326	-0.9988	0.0419
**4.**	3.9811	0.2204	-4.8362	0.3087	-0.9939	0.0976
**5.**	4.5517	0.1316	-5.1442	0.1843	-0.9981	0.0583
**6.**	4.1987	0.1299	-5.0042	0.1819	-0.998	0.0575
**7.**	2.585	0.0981	-3.5552	0.1374	-0.9978	0.0435
**8.**	3.286	0.2234	-4.3126	0.3128	-0.9922	0.0989
**9.**	3.8166	0.1674	-4.6936	0.2345	-0.9963	0.0741
**10.**	4.9755	0.2587	-5.6853	0.3412	-0.9964	0.0763
**11.**	4.3383	0.1302	-5.3142	0.1823	-0.9982	0.0577
**12.**	3.274	0.1584	-4.2238	0.2218	-0.9959	0.0701
**13.**	3.9604	0.1618	-4.9279	0.2265	-0.9968	0.0716
**14.**	4.1461	0.1276	-5.047	0.1787	-0.9981	0.0565
**15.**	4.1306	0.1815	-5.1283	0.2542	-0.9963	0.0804
**16.**	5.3221	0.1461	-6.19	0.2046	-0.9984	0.0647
**17.**	3.9331	0.1246	-4.8573	0.1745	-0.9981	0.0552
**18.**	4.1865	0.2298	-5.1232	0.3218	-0.9941	0.1018

[1] - $S(R_{M0})$ – standard error of the intercept; S(S) – standard error of the slope of the regression line; r – correlation coefficient; Se – standard error of the estimation

Table S2. R_{Mw}, S parameters and statistics[1] obtained by C-18/ACN HPTLC .

No.	R_{Mw}	$S(R_{Mw})$	S	S(S)	r	Se
**1.**	2.3065	0.2249	-3.3042	0.3346	-0.9801	0.14
**2.**	2.5427	0.2273	-3.4423	0.3382	-0.9812	0.1415
**3.**	2.5075	0.2328	-3.3828	0.3463	-0.9797	0.1449
**4.**	2.2474	0.1916	-3.1177	0.2851	-0.9837	0.1193
**5.**	2.6662	0.1763	-3.3255	0.2623	-0.9878	0.1097
**6.**	2.8733	0.2099	-3.951	0.3123	-0.9877	0.1306
**7.**	1.2338	0.1251	-2.6639	0.2029	-0.9914	0.0903
**8.**	1.8644	0.1694	-3.4601	0.2749	-0.9907	0.0869
**9.**	2.2408	0.1537	-3.3807	0.2286	-0.991	0.0957
**10.**	3.154	0.1881	-4.0979	0.2798	-0.9908	0.1171
**11.**	3.022	0.1969	-4.3598	0.293	-0.9911	0.1226
**12.**	2.2086	0.1163	-3.5864	0.1731	-0.9954	0.0724
**13.**	2.3586	0.1684	-3.5546	0.2506	-0.9902	0.1048
**14.**	2.5124	0.1936	-3.6927	0.2881	-0.988	0.1205
**15.**	2.4307	0.1847	-3.5697	0.2748	-0.9884	0.115
**16.**	3.1284	0.2174	-4.1169	0.3235	-0.9879	0.1353
**17.**	2.7075	0.1595	-4.0199	0.2373	-0.9931	0.0993
**18.**	2.6933	0.1841	-3.7917	0.2739	-0.9897	0.1146

[1] - $S(R_{M0})$ – standard error of the intercept; S(S) – standard error of the slope of the regression line; r – correlation coefficient; Se – standard error of the estimation

Table S3. R_{Mw}, S parameters and statistics[1] obtained by C-18/acetone HPTLC.

No.	R_{Mw}	$S(R_{Mw})$	S	S(S)	r	Se
**1.**	3.0688	0.3906	-4.145	0.594	-0.9705	0.1328
**2.**	3.4917	0.4576	-4.8177	0.6958	-0.9701	0.1556
**3.**	3.462	0.4268	-4.865	0.649	-0.9743	0.1451
**4.**	3.1554	0.3994	-4.5141	0.6073	-0.9739	0.1358
**5.**	4.2145	0.5956	-5.7936	0.9057	-0.9652	0.2025
**6.**	4.1087	0.5162	-5.7083	0.785	-0.9728	0.1755
**7.**	1.9255	0.3551	-3.0554	0.54	-0.9562	0.1207
**8.**	2.3669	0.3157	-3.6686	0.4801	-0.9753	0.1073
**9.**	3.2825	0.2769	-4.6956	0.4211	-0.9882	0.0942
**10.**	5.1557	0.4453	-6.955	0.6771	-0.9861	0.1514
**11.**	4.4748	0.3946	-6.1749	0.6001	-0.9861	0.1342

**12.**	3.0103	0.3035	-4.392	0.4615	-0.9838	0.1032
**13.**	3.3822	0.4698	-4.8625	0.7144	-0.9691	0.1597
**14.**	3.4987	0.3418	-5.0132	0.5197	-0.9843	0.1162
**15.**	3.2517	0.3671	-4.7076	0.5582	-0.9796	0.1248
**16.**	4.4732	0.2694	-6.0637	0.4096	-0.9932	0.0916
**17.**	3.6933	0.4166	-5.2445	0.6334	-0.9788	0.1416
**18.**	3.0688	0.3906	-4.145	0.594	-0.9705	0.1328

[1] - $S(R_{M0})$ – standard error of the intercept; S(S) – standard error of the slope of the regression line; r – correlation coefficient; Se – standard error of the estimation

Table S4. R_{Mw}, S parameters and statistics[1] obtained by C-18/1.4-dioxane HPTLC.

No.	R_{Mw}	$S(R_{Mw})$	S	S(S)	r	Se
**1.**	2.8576	0.2354	-4.6743	0.4212	-0.988	0.0942
**2.**	3.5155	0.1865	-5.6205	0.3336	-0.9948	0.0746
**3.**	3.525	0.1642	-5.6349	0.2938	-0.9959	0.0657
**4.**	3.3997	0.1911	-5.4845	0.3419	-0.9942	0.0764
**5.**	4.3974	0.1317	-6.6904	0.2356	-0.9981	0.0527
**6.**	4.185	0.0946	-6.4282	0.1693	-0.999	0.0379
**7.**	2.1331	0.4042	-3.9727	0.723	-0.9537	0.1617
**8.**	2.2823	0.3027	-4.0903	0.5414	-0.9747	0.1211
**9.**	3.19	0.1886	-5.2849	0.3374	-0.9939	0.0754
**10.**	4.6118	0.0753	-6.7659	0.1272	-0.9996	0.0188
**11.**	4.6691	0.3336	-7.1249	0.5968	-0.9896	0.1334
**12.**	2.7468	0.1897	-4.6552	0.3394	-0.9921	0.0759
**13.**	3.1553	0.1478	-5.1508	0.2643	-0.9961	0.0591
**14.**	3.4087	0.1967	-5.6106	0.352	-0.9941	0.0787
**15.**	3.5901	0.1793	-5.8171	0.3208	-0.9955	0.0717
**16.**	4.4149	0.13	-6.6916	0.2325	-0.9982	0.052
**17.**	3.7259	0.1599	-5.972	0.286	-0.9966	0.064
**18.**	3.6674	0.2179	-5.7207	0.3899	-0.9931	0.0872

[1] - $S(R_{M0})$ – standard error of the intercept; S(S) – standard error of the slope of the regression line; r – correlation coefficient; Se – standard error of the estimation

Table S5. R_{Mw}, S parameters and statistics[1] obtained by C-8/methanol HPTLC.

No.	R_{Mw}	$S(R_{Mw})$	S	S(S)	r	Se
**1.**	3.2301	0.2909	-4.2198	0.4073	-0.9863	0.1288
**2.**	3.7659	0.2011	-4.7853	0.2816	-0.9948	0.089

**3.**	3.768	0.195	-4.7961	0.273	-0.9952	0.0863
**4.**	3.5679	0.4222	-4.6146	0.5911	-0.9763	0.1869
**5.**	4.0852	0.2885	-5.1206	0.404	-0.9908	0.1278
**6.**	4.2843	0.2662	-5.5348	0.3727	-0.9933	0.1179
**7.**	2.2919	0.1874	-3.4296	0.2624	-0.9913	0.083
**8.**	2.9989	0.2293	-4.1905	0.321	-0.9913	0.1015
**9.**	3.3875	0.1029	-4.4726	0.1442	-0.9984	0.0456
**10.**	4.2991	0.0391	-5.3897	0.0515	-0.9999	0.0115
**11.**	4.7993	0.4716	-6.153	0.6604	-0.9832	0.2088
**12.**	3.0868	0.175	-4.2396	0.2451	-0.995	0.0775
**13.**	3.6035	0.2573	-4.7723	0.3602	-0.9916	0.1139
**14.**	4.2483	0.3498	-5.5317	0.4898	-0.9884	0.1549
**15.**	3.8757	0.277	-5.081	0.3878	-0.9914	0.1226
**16.**	4.8401	0.3088	-6.061	0.4324	-0.9925	0.1368
**17.**	3.7409	0.26	-4.9394	0.3641	-0.9919	0.1152
**18.**	4.101	0.2772	-5.1822	0.3881	-0.9917	0.1227

[1] - $S(R_{M0})$ – standard error of the intercept; S(S) – standard error of the slope of the regression line; r – correlation coefficient; Se – standard error of the estimation

Table S6. R_{Mw}, S parameters and statistics[1] obtained by C-8/ACN HPTLC.

**No.**	R_{Mw}	$S(R_{Mw})$	S	S(S)	r	Se
**1.**	2.4247	0.1654	-3.9657	0.2683	-0.9932	0.0849
**2.**	2.6218	0.198	-4.1234	0.3212	-0.991	0.1016
**3.**	2.5355	0.1569	-4.0074	0.2545	-0.994	0.0805
**4.**	2.4741	0.137	-3.9675	0.2222	-0.9953	0.0703
**5.**	2.8428	0.2078	-4.3194	0.3371	-0.991	0.1066
**6.**	2.9276	0.147	-4.6523	0.2385	-0.9961	0.0754
**7.**	1.5285	0.1575	-3.3688	0.2555	-0.9915	0.0808
**8.**	2.0612	0.0678	-4.0361	0.11	-0.9989	0.0348
**9.**	2.6101	0.1667	-4.5846	0.2704	-0.9948	0.0855
**10.**	3.3915	0.2428	-5.1579	0.3939	-0.9914	0.1246
**11.**	3.2135	0.1217	-5.1249	0.1974	-0.9978	0.0624
**12.**	2.5055	0.0627	-4.456	0.1017	-0.9992	0.0322
**13.**	2.62	0.0685	-4.4417	0.1112	-0.9991	0.0352
**14.**	2.7387	0.1013	-4.5844	0.1643	-0.9981	0.052
**15.**	2.6766	0.0835	-4.4186	0.1355	-0.9986	0.0429
**16.**	3.3186	0.1646	-5.0415	0.267	-0.9958	0.0844
**17.**	2.8906	0.0963	-4.8089	0.1562	-0.9984	0.0494
**18.**	2.9122	0.1826	-4.5548	0.2963	-0.9937	0.0937

[1] - S(RM0) – standard error of the intercept; S(S) – standard error of the slope of the regression line; r – correlation coefficient; Se – standard error of the estimation

Table S7. R_{Mw}, S parameters and statistics[1] obtained by C-8/acetone HPTLC.

No.	R_{Mw}	S(R_{Mw})	S	S(S)	r	Se
**1.**	2.8085	0.3002	-4.1665	0.5175	-0.9776	0.1246
**2.**	3.451	0.3455	-5.2622	0.5957	-0.9813	0.1435
**3.**	3.1503	0.2377	-4.862	0.4098	-0.9895	0.0987
**4.**	3.2056	0.2501	-5.0061	0.4311	-0.9891	0.1038
**5.**	3.8806	0.2954	-5.8501	0.5092	-0.9888	0.1226
**6.**	3.9255	0.2895	-5.9535	0.499	-0.9896	0.1202
**7.**	1.8372	0.2008	-3.2382	0.3461	-0.9833	0.0834
**8.**	2.2082	0.2074	-3.7608	0.3574	-0.9867	0.0861
**9.**	3.6	0.2992	-5.6021	0.5158	-0.9875	0.1242
**10.**	4.6015	0.4359	-6.6585	0.7065	-0.9889	0.1045
**11.**	4.4566	0.3503	-6.5978	0.604	-0.9877	0.1455
**12.**	2.9359	0.2093	-4.6341	0.3608	-0.991	0.0869
**13.**	3.1658	0.2	-4.9489	0.3447	-0.9928	0.083
**14.**	3.6193	0.2668	-5.6131	0.4599	-0.9901	0.1108
**15.**	3.3827	0.2305	-5.2874	0.3974	-0.9916	0.0957
**16.**	4.5467	0.2183	-6.6434	0.3764	-0.9952	0.0906
**17.**	3.81	0.265	-5.8383	0.4568	-0.9909	0.11
**18.**	3.6	0.2992	-5.6021	0.5158	-0.9875	0.1242

[1] - S(RM0) – standard error of the intercept; S(S) – standard error of the slope of the regression line; r – correlation coefficient; Se – standard error of the estimation

Table S8. R_{Mw}, S parameters and statistics[1] obtained by C-8/1.4-dioxane HPTLC.

No.	R_{Mw}	S(R_{Mw})	S	S(S)	r	Se
**1.**	2.7718	0.2043	-4.618	0.3655	-0.9907	0.0817
**2.**	3.1266	0.2078	-5.0533	0.3718	-0.992	0.0831
**3.**	3.0505	0.1914	-4.945	0.3424	-0.9929	0.0766
**4.**	3.0437	0.1938	-4.9879	0.3467	-0.9928	0.0775
**5.**	3.6609	0.2007	-5.6367	0.359	-0.994	0.0803
**6.**	3.7337	0.2305	-5.804	0.4124	-0.9925	0.0922
**7.**	1.7267	0.2476	-3.4185	0.4429	-0.9757	0.099
**8.**	1.9132	0.3183	-3.5051	0.5693	-0.9626	0.1273
**9.**	3.049	0.2206	-5.0986	0.3947	-0.9911	0.0883
**10.**	4.2828	0.2865	-6.4647	0.5125	-0.9907	0.1146

**11.**	3.8724	0.2363	-5.9527	0.4227	-0.9925	0.0945
**12.**	2.2166	0.3215	-3.6629	0.5751	-0.965	0.1286
**13.**	2.9949	0.2179	-4.961	0.3898	-0.9909	0.0872
**14.**	3.3271	0.2439	-5.5135	0.4362	-0.9907	0.0975
**15.**	3.3429	0.2397	-5.4854	0.4288	-0.991	0.0959
**16.**	4.1483	0.2647	-6.3564	0.4735	-0.9918	0.1059
**17.**	3.4709	0.2051	-5.6229	0.3669	-0.9937	0.082
**18.**	3.5733	0.1777	-5.6858	0.3179	-0.9953	0.0711

[1] - $S(R_{M0})$ – standard error of the intercept; S(S) – standard error of the slope of the regression line; r – correlation coefficient; Se – standard error of the estimation

Table S9. Values of the lipophilicity descriptors determined by HPLC (log k_w) and calculated with ChemDraw Ultra ver. 10.0 (log P, Clog P). and Marvin ver. 19.9 (log P Cons) software for investigated compounds.

No.	log k_w C18/Met [1]	log k_w IAM [1]	log k_w C8/Met [1]	log P [2]	ClogP [2]	log P Cons [3]
**1.**	2.537	2.48	3.674	3.86	2.54	3.19
**2.**	3.000	2.837	4.266	4.34	3.04	3.7
**3.**	3.002	2.837	4.341	4.01	**2.69**	3.33
**4.**	2.670	2.812	4.13	4.41	3.01	3.79
**5.**	3.678	3.337	4.691	4.97	3.72	4.4
**6.**	3.690	3.156	5.07	4.78	3.43	4.07
**7.**	1.907	2.562	2.98	3.08	1.86	2.58
**8.**	2.545	2.539	3.325	3.34	1.99	2.73
**9.**	2.512	2.603	4.045	-	2.29	3.13
**10.**	3.734	3.318	5.517	5.94	4.1	5.23
**11.**	2.477	3.227	5.489	5.59	3.33	4.24
**12.**	2.986	2.525	3.862	4.25	3.41	3.29
**13.**	3.005	2.813	4.428	4.74	3.9	3.8
**14.**	4.296	3.269	4.58	4.41	3.95	3.85
**15.**	3.238	3.074	4.687	5.35	4.58	4.28
**16.**	3.059	3.67	5.351	5.71	5.12	4.85
**17.**	2.780	2.949	4.395	4.97	3.96	4.04
**18.**	2.879	2.92	4.48	4.08	3.4	3.25

Table S10. Relevant component characteristics of PCA analysis of HPTLC chromatographic data obtained. Decomposition based on correlation matrix. Calculations were performed using JASP Team (2023) [4].

	unrotated solution	rotated solution

Component	eigenvalue	proportion var.	cumulative	sum sq. loadings	proportion var.	cumulative
Component 1	7.512	0.9390	0.9390	7.512	0.9390	0.9390
Component 2	0.1520	0.01900	0.9580	0.1520	0.01900	0.9580

Table S11. Component loadings for respective chromatographic systems. Calculations were performed using JASP Team (2023) [4].

System	**PC1**	**PC2**	**Uniqueness**
R_{Mw} C8/ACE	0.982	0.031	0.035
R_{Mw} C8/Dx	0.981	-0.026	0.036
R_{Mw} C8/ACN	0.979	0.004	0.041
R_{Mw} C18/ACN	0.979	-0.014	0.042
R_{Mw} C18/Dx	0.970	0.103	0.049
R_{Mw} C8/Met	0.956	-0.223	0.036
R_{Mw} C18/ACE	0.953	0.266	0.022
R_{Mw} C18/Met	0.952	-0.137	0.075

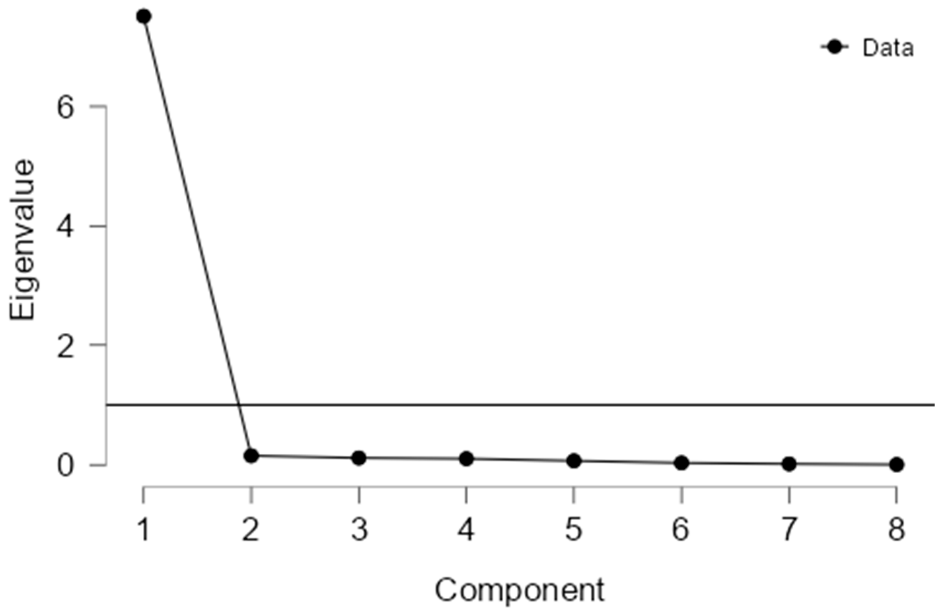

Figure S1. Scree plot summarizing performed PCA analysis. Drawn using ASP Team (2023) [4].

[1] Studzinski, M.; Baranska, K.; Paw, B.; Senczyna, B.; Paszko, T.; Matysiak, J., In silico and RP HPLC studies of biologically active 1,3,4-thiadiazol-2-yl)-benzene-1,3-diols. *Molecules* **2025**, *30*, (19).
[2] ChemDraw Ultra 10.0. Cambridge Soft Corporation, Cambridge, USA 2006.
[3] Marvin, version 19.9, ChemAxon Ltd, 2024.
[4] JASP, version 0.17.2.1; JASP Team: Amsterdam, The Netherlands, 2023.
